# Building a knowledge graph to enable precision medicine

**DOI:** 10.1038/s41597-023-01960-3

**Published:** 2023-02-02

**Authors:** Payal Chandak, Kexin Huang, Marinka Zitnik

**Affiliations:** 1 Harvard-MIT Program in Health Sciences and Technology, Cambridge, MA 02139 USA; 2 Department of Computer Science, Stanford University, Stanford, CA 94305 USA; 3 Department of Biomedical Informatics, Harvard Medical School, Harvard University, Boston, MA 02115 USA; 4 Broad Institute of MIT and Harvard, Cambridge, MA 02142 USA; 5 Harvard Data Science Initiative, Cambridge, MA 02138 USA

**Keywords:** Data integration, Network topology, Predictive medicine, Medical research, Machine learning

## Abstract

Developing personalized diagnostic strategies and targeted treatments requires a deep understanding of disease biology and the ability to dissect the relationship between molecular and genetic factors and their phenotypic consequences. However, such knowledge is fragmented across publications, non-standardized repositories, and evolving ontologies describing various scales of biological organization between genotypes and clinical phenotypes. Here, we present PrimeKG, a multimodal knowledge graph for precision medicine analyses. PrimeKG integrates 20 high-quality resources to describe 17,080 diseases with 4,050,249 relationships representing ten major biological scales, including disease-associated protein perturbations, biological processes and pathways, anatomical and phenotypic scales, and the entire range of approved drugs with their therapeutic action, considerably expanding previous efforts in disease-rooted knowledge graphs. PrimeKG contains an abundance of ‘indications’, ‘contradictions’, and ‘off-label use’ drug-disease edges that lack in other knowledge graphs and can support AI analyses of how drugs affect disease-associated networks. We supplement PrimeKG’s graph structure with language descriptions of clinical guidelines to enable multimodal analyses and provide instructions for continual updates of PrimeKG as new data become available.

## Abstract (summary)

**Table:** 

Measurement(s)	knowledge graph • Relation Code • textual entity
Technology Type(s)	machine learning • computational modeling technique

## Background & Summary

Precision medicine takes an approach to disease diagnosis and treatment that accounts for the variability in genetics, environment, and lifestyle across individuals^1^. To be precise, medicine must revolve around data and learn from biomedical knowledge and health information^2^. Nevertheless, many barriers to efficiently exploiting information across biological scales slow down the research and development of individualized care^2^. While many have acknowledged the difficulties in linking biomedical knowledge to patient-level health information^2–5^, few realize that biomedical knowledge is itself fragmented. Biomedical knowledge about complex diseases comes from different organizational scales, including genomics, transcriptomics, proteomics, molecular functions, intra- and inter-cellular pathways, phenotypes, therapeutics, and environmental effects. For any given disease, information from these organizational scales is scattered across publications, non-standardized data repositories, evolving ontologies, and clinical guidelines. Developing networked relationships between these sources can support research in precision medicine.

A resource that comprehensively describes the relationships of diseases to biomedical entities would enable systematic study of human disease. Understanding the connections between diseases, drugs, phenotypes, and other entities could open the doors for many types of research, including but not limited to the study of phenotyping^6–8^, disease etiology^9^, disease similarity^10^, diagnosis^11–13^, treatments^14^, drug-disease relationships^15–17^, mechanisms of drug action^18^ and resistance^3^, drug repurposing^19–21^, drug discovery^22,23^, adverse events^24,25^, and combination therapies^26^. Knowledge graphs developed for individual diseases have yielded insights into respective disease areas^27–42^. Nevertheless, the costs and extended timelines of these individual efforts point to a need for a resource that would unify biomedical knowledge and enable the investigation of diseases at scale.

While many primary data resources contain information about diseases, consolidating them into a comprehensive, disease-rich, and functional knowledge graph presents three challenges. First, existing approaches to network analysis of diseases require expert review and curation of data in the knowledge graph^29,30,43^. While incredibly detailed, such efforts require substantial manual labor and expensive expert input, making them difficult to scale. Second, there lacks a consistent representation of diseases across biomedical datasets and clinical guidelines. Rather than have a standardized disease ontology, database developers select the ontology that best suits their function from a multitude of biorepositories^44–54^. Because each set of disease vocabulary was tailored for some to serve a unique purpose, their disease encodings overlap unsystematically and are often in conflict. For instance, International Classification of Diseases (ICD) codes^50^ are optimized for medical billing whereas MedGen^53^, PhenoDB^51^, and Orphanet^48^ focus on rare and genetic diseases. Moreover, expertly curated disease descriptions in medical repositories do not follow any naming conventions^48,55^. The lack of standardized disease representations and the multimodal nature of the datasets makes it challenging to harmonize biomedical knowledge at scale. Third, the definition of diseases as discrete and distinct units of analysis remains medically and scientifically ambiguous. For instance, while autism spectrum disorder is considered a medical diagnosis, the condition has many subtypes linked to clinically divergent manifestations^56,57^. Clinically studied disease subtypes often do not correlate clearly with those defined in disease ontologies. Although only three subtypes of autism have been clinically identified^57^, the Unified Medical Language System (UMLS)^46^ describes 192 subtypes, the Monarch Disease Ontology (MONDO)^44^ describes 37 subtypes, and finally, Orphanet^48^ contains 6 disease entries for autism. The challenge in reconciling disease entities is only exacerbated by the variety of synonyms and abbreviations available for any particular disease^58^ and the difficulty in linking structured disease entities to unstructured names in text^59^. Meaningful disease entity resolution across multimodal, non-standardized datasets is critical for developing resources useful for precision medicine tasks.

While drug repurposing remains the focus of knowledge graph development^33,37,39,42,60–62^, considerable effort has been devoted to building knowledge graphs from biomedical literature^28,31,40^ and clinical records^29,30,34,63^. As early efforts to investigate the connection between clinical manifestations of diseases and their underlying molecular interactions, the Human diseases network (HDN) and Human symptoms-disease network (HSDN) have been influential in demonstrating the relevance of disease-centric knowledge graphs^64,65^. Scalable Precision Medicine Open Knowledge Engine (SPOKE) network is a seminal effort that linked many heterogeneous biomedical databases to build a knowledge graph focused on diseases^38^. Although SPOKE is limited to 137 diseases and lacks multimodal connections between textual clinical guidelines and tabular molecular data, it has enabled many precision medicine efforts, including overlaying individual patient information onto the SPOKE’s graph^35^. Another knowledge graph focused exclusively on rare diseases, Genetic and Rare Diseases Information Center (GARD)^34^, has advanced understanding of unmet medical needs and evidence-based studies for patients with under-diagnosed diseases^66,67^. Most recently, a White House initiative led the development of the COVID-19 Open Research Dataset (CORD-19)^68^. CORD-19 is designed to empower data-driven medicine during the pandemic by powering neural search engines for healthcare workers^69,70^ and providing insights into drug repurposing opportunities^71^. Collectively, biomedical knowledge graphs have lent themselves to a variety of scientific discoveries^72,73^, methodological innovations^74–76^ and coordinated initiatives for model evaluation and benchmarking^32,36,77^. Further, knowledge graphs facilitated research across various problems faced by the biomedical community. Nevertheless, due to the medical heterogeneity of diseases, the multimodal nature of disease information, and the incompatibility of existing disease repositories, knowledge graphs focused on diseases have not yet achieved the scale or impact of biomedical efforts.

**Fig. 1:**
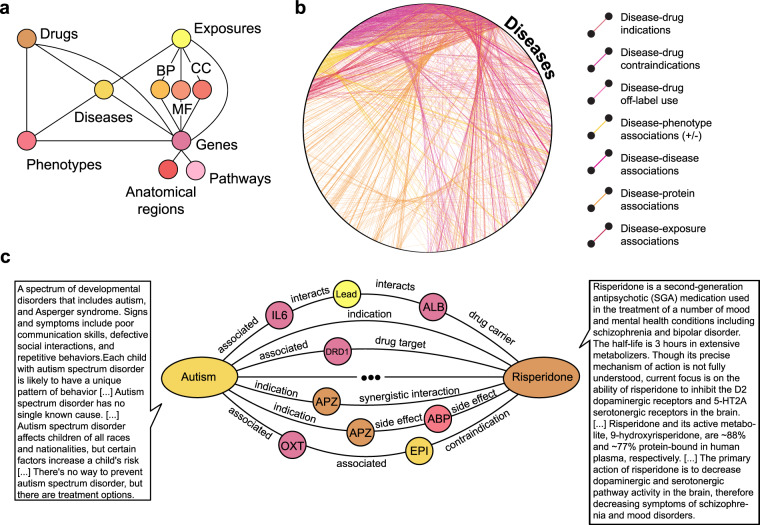
Overview of PrimeKG multimodal knowledge graph. (**a**) Shown is a schematic overview of the various types of nodes in PrimeKG and the relationships they have with other nodes in the graph. (**b)** All disease nodes in PrimeKG shown in a circular layout together with disease-associated information. All relationships between disease nodes and any other node type are depicted here. Disease nodes are densely connected to four other node types in PrimeKG through seven types of relations. (**c)** Shown is an example of paths in PrimeKG between the disease node ‘Autism’ and the drug node ‘Risperidone’. Intermediate nodes are colored by their node type from panel a. We also display snippets of text features for both nodes to demonstrate the multimodality of PrimeKG. Abbreviations - MF: molecular function, BP: biological process, CC: cellular component, APZ: Apiprazole, EPI: epilepsy, ABP: abdominal pain, +/− associations: positive and negative associations.

Precision Medicine Knowledge Graph (PrimeKG) is a knowledge graph providing a holistic and multimodal view of diseases. We integrate 20 high-quality resources, biorepositories, and ontologies to curate this knowledge graph. Across 129,375 nodes and 4,050,249 relationships, PrimeKG captures information on ten major biological scales, including disease-associated perturbations in the proteome, biological processes, molecular pathways, anatomical and phenotypic scales, environmental exposures, and the range of approved and experimental drugs together with their therapeutic action (Fig. 1a,b). We demonstrate that PrimeKG improves on coverage of diseases, both rare and common, by one-to-two orders of magnitude compared to existing knowledge graphs. Moreover, disease nodes in PrimeKG are densely connected to many other node types, including phenotypes, exposures, and drugs. We tune PrimeKG specifically to support artificial intelligence analyses to understand how drugs target disease-associated molecular perturbations by including an abundance of ‘indications’, ‘contradictions’, and ‘off-label use’ drug-disease edges, which are usually missing or sparse in other knowledge graphs. We supplement PrimeKG’s graph structure with textual descriptions of clinical guidelines for drug and disease nodes to enable multimodal analyses (Fig. 1c). Finally, we address the disease entity resolution challenge by improving the correspondence between diseases in PrimeKG and disease subtypes found in the clinic to enable PrimeKG-powered analyses in precision medicine.

## Methods

We proceed with a detailed description of the 20 primary data resources used to build PrimeKG. Table 1 lists primary data resources organized by node types in PrimeKG. Since many resources contain information about multiple types of nodes in PrimeKG, we ordered these resources alphabetically in the following description.

**Table 1:** Information about nodes and node types in PrimeKG.

Node Type	Count	Percent (%)	Data Sources
Biological process	28,642	22.1	CTD, Entrez Gene, Gene Ontology
Protein	27,671	21.4	Bgee, CTD, DisGeNET, DrugBank, Entrez Gene, Human Phenotype Ontology, Human PPI Network, Reactome, UMLS
Disease	17,080	13.2	CTD, DisGeNET, Disease Ontology, Drug Central, Human Phenotype Ontology, Mayo Clinic, MONDO Disease Ontology, Orphanet
Phenotype	15,311	11.8	DisGeNET, Human Phenotype Ontology, SIDER
Anatomy	14,035	10.8	Bgee, UBERON
Molecular function	11,169	8.6	CTD, Entrez Gene, Gene Ontology
Drug	7,957	6.2	DrugBank, Drug Central, SIDER
Cellular component	4,176	3.2	CTD, Entrez Gene, Gene Ontology
Pathway	2,516	1.9	Reactome
Exposure	818	0.6	CTD
Total	129,375	100.0	20 primary data sources

### A. Overview of primary data resources

**Fig. 2:**
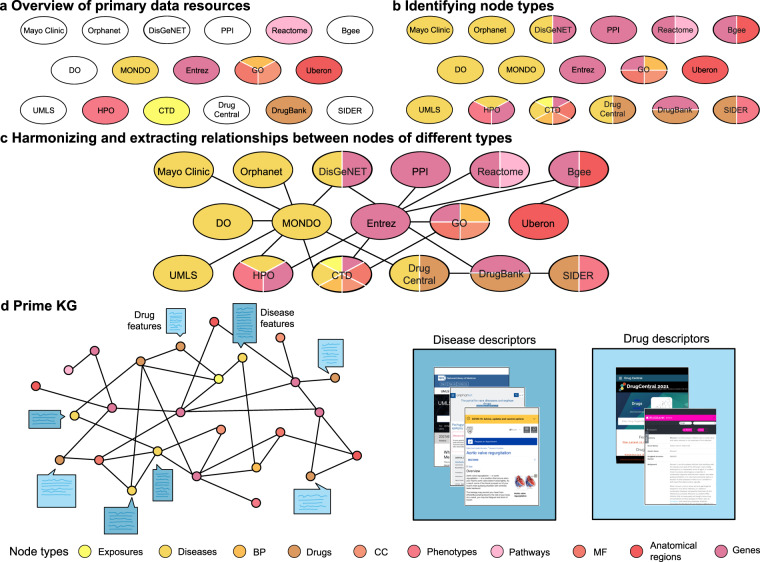
Building PrimeKG. The panels sequentially illustrate the process of developing the Precision Medicine Knowledge Graph. (**a)** Shown are 20 primary data resources curated to develop PrimeKG. The colors highlight which data records are used to uniquely identify each node type. For example, GO is colored by biological processes, cellular components, and molecular functions because GO terms are the unique identifiers used to define nodes for these three node types. (**b)** Primary resources are colored by each node type for which they possess information. For example, GO provides links from biological processes, cellular components, and molecular functions to genes. As a result, we add the fourth color to represent the gene/protein class. (**c)** Illustrated is the process of harmonizing these primary data records to extract relationships between node types. (**d)** The left side illustrates PrimeKG, and the right side shows all the textual sources of clinical information on drugs and diseases. The node type legend is consistent across the figure. Abbreviations - MF: molecular function, BP: biological process, CC: cellular component, PPI: protein-protein interactions, DO: disease ontology, MONDO: MONDO disease ontology, Entrez: Entrez gene, GO: gene ontology, UMLS: unified medical language system, HPO: human phenotype ontology, CTD: comparative toxicogenomics database, SIDER: side effect resource.

To develop a comprehensive knowledge graph to study diseases, we considered 20 primary resources and a number of additional repositories of biological and clinical information. Figure 2a provides an overview of all 20 resources. The data resources provide widespread coverage of biomedical entities, including proteins, genes, drugs, diseases, anatomy, biological processes, cellular components, molecular functions, exposures, disease phenotypes and drug side effects. These were high-quality datasets, either expertly curated annotations such as the Disease Gene Network (DisGeNet) of gene-disease associations^78^ and the Mayo Clinic knowledgebase^55^, widely-used standardized ontologies such as the MONDO Disease Ontology^44^, or direct readouts of experimental measurements such as Bgee gene expression knowledgebase^79^ and DrugBank^80^. A complete list of primary resources and the processing steps are listed in the Methods section.

#### Bgee gene expression knowledge base in animals

Bgee^79^ contains gene expression patterns across multiple animal species. We retrieved gene expression data for humans from ftp://ftp.bgee.org/current/download/calls/expr_calls/Homo_sapiens_expr_advanced.tsv.gz on 31 May 2021. First, we ensured that all anatomical entities were coded using the UBERON ontology. Then, we filtered the dataset to retain gold quality calls with a false discovery rate corrected p-value of ≤ 0.01. Finally, we filtered the expression rank column to extract highly expressed genes. Based on the range and distribution of the expression rank, we retained all data with an expression rank less than 25,000. After processing, we had 1,786,311 anatomy-protein associations where gene expression was found to be present or absent.

#### Comparative toxicogenomics database

The Comparative Toxicogenomics Database (CTD)^81^ is focused on the impact of environmental exposures on human health. We retrieved information about exposures (05/21 version) from http://ctdbase.org/reports/CTD_exposure_events.csv.gz on 9 Jun 2021. Processing involved removing header comments from the raw file. After processing, our data contained 180,976 associations of exposures with exposures, proteins, diseases, biological processes, molecular functions, and cellular components.

#### DisGeNET knowledgebase of gene-disease associations

DisGeNET^78^ is a resource about the relationships between genes and human disease that has been curated by experts. We retrieved curated disease-gene associations (version 7.0) from https://www.disgenet.org/static/disgenet_ap1/files/downloads/curated_gene_disease_associations.tsv.gz on 31 May 2021. The raw data file, ‘curated_gene_disease_associations.tsv’ was not processed further and contained 84,038 associations of genes with diseases and phenotypes.

#### Disease ontology

Disease Ontology^47^ groups diseases in many meaningful clusters using clinically relevant characteristics. For instance, diseases are grouped by the anatomical entity. We retrieved the ontology from https://raw.githubusercontent.com/DiseaseOntology/HumanDiseaseOntology/main/src/ontology/HumanDO.obo on 29 Jun 2021. The raw data ‘HumanDO.obo’ is mapped to disease nodes in PrimeKG. As the MONDO Disease Ontology is not organized anatomically or by clinical specialty, the include of Disease Ontology in PrimeKG allows users of PrimeKG to explore disease nodes in a medically meaningful format.

#### DrugBank

DrugBank^80^ is a resource that contains pharmaceutical knowledge. We retrieved the knowledgebase (version 5.1.8) from https://go.drugbank.com/releases/5-1-8/downloads/all-full-database on 31 May 2021. Processing involved using the Beautiful Soup package^82^ to extract synergistic drug interactions. The processed data contains 2,682,157 associations. We also extracted drug features from the raw data. For over 14,000 drugs, we construct 12 drug features, including group, state, description, mechanism of action, Anatomical Therapeutic Chemical (ATC) code, pharmacodynamics, half-life, target protein binding information, and pathways.

We also retrieved information about drug targets from https://go.drugbank.com/releases/5-1-8/downloads/target-all-polypeptide-ids, about drug enzymes from https://go.drugbank.com/releases/5-1-8/downloads/enzyme-all-polypeptide-ids, about drug carriers from https://go.drugbank.com/releases/5-1-8/downloads/carrier-all-polypeptide-ids, about drug transporters from https://go.drugbank.com/releases/5-1-8/downloads/transporter-all-polypeptide-ids all on 31 May 2021. Processing involved combining all four resources and mapping gene names from UniProt IDs to the National Center for Biotechnology Information (NCBI) gene IDs using vocabulary retrieved from Human Gene Nomenclature (HGNC) gene names https://www.genenames.org. The processed data contains 26,118 drug-protein interactions.

#### Drug central

Drug Central^83^ is a resource that curates information about drug-disease interactions. We retrieved the Drug Central SQL database from https://drugcentral.org/ActiveDownload on 1 Jun 2021. The database was loaded into Postgres SQL, and drug-disease relationships were extracted. The processed data contains 26,698 indication edges, 8,642 contraindication edges, and 1,917 off-label use edges. We also extracted drug features from the Drug Central SQL database from the ‘structures’ and ‘structure_type’ tables. We extracted features for over 4,500 drugs, representing each drug with features including topological polar surface area (TPSA), molecular weight, and cLogP, which is the logarithm of a compound’s partition coefficient between n-octanol and water, and is a well established measure of the compound’s hydrophilicity. For example, the features for *Atorvastatin* are organic structure, the molecular weight of 558.65, TPSA of 111.79 and the cLogP value of 4.46.

#### Entrez gene

Entrez Gene^84^ is a resource maintained by the NCBI that contains vast amounts of gene-specific information. We retrieved data about relations between genes and Gene Ontology terms from https://ftp.ncbi.nlm.nih.gov/gene/DATA/gene2go.gz on 31 May 2021. Processing involved using the GOATOOLS package^85^ to extract relations between genes and Gene Ontology terms. The processed data contains 297,917 associations of genes with biological processes, molecular functions, and cellular components.

#### Gene ontology

The Gene Ontology^86^ describes molecular functions, cellular components, and biological processes. We retrieved the ontology from http://purl.obolibrary.org/obo/go/go-basic.obo on 31 May 2021. Processing involved using the GOATOOLS package^85^ to extract information for Gene Ontology terms and relations between GO terms. The processed data contains 71,305 hierarchical associations between biological processes, molecular functions, and cellular components.

#### Human phenotype ontology

The Human Phenotype Ontology^45^ (version hpo-obo@2021-04-13) provides information on phenotypic abnormalities found in diseases. We retrieved the ontology from http://purl.obolibrary.org/obo/hp.obo on 31 May 2021. Processing involved parsing the ontology file to extract phenotype terms in the ontology, parent-child relationships, and cross-references to other ontologies. The processed data contains disease-phenotype, protein-phenotype, and phenotype-phenotype edges. We also retrieved expertly curated annotations from http://purl.obolibrary.org/obo/hp/hpoa/phenotype.hpoa on 31 May 2021. From this curated source, we extracted 218,128 positive and negative associations between diseases and phenotypes.

#### Mayo clinic

Mayo Clinic is a nonprofit academic medical center and biomedical research institution care^55^. It maintains a knowledgebase, https://www.mayoclinic.org/diseases-conditions, with information about symptoms, causes, risk factors, complications, and prevention of 2,227 diseases and conditions. We web-scraped the knowledgebase and extracted descriptions for these diseases and conditions using the *mayo.py* and *diseases.py* scripts on 28 March 2021. The raw data is available at ‘mayo.csv’ in the PrimeKG repository.

For example, we illustrate the extracted features for atrial fibrillation from Mayo Clinic. ‘Some people with atrial fibrillation have no symptoms […] others may experience signs and symptoms such as Palpitations, Weakness, […] and Chest Pain. The disease occurs when ‘the two upper chambers of your heart experience chaotic electrical signals […] As a result, they quiver. The AV node is bombarded with impulses to get through to the ventricles. Certain factors may increase your risk of developing atrial fibrillation, including age, heart disease, […] and obesity. Complications include: ‘the chaotic rhythm causing blood to pool in your atria and form clots […] leading to a stroke. […] Atrial fibrillation, especially if not controlled, may weaken the heart and lead to heart failure. To prevent atrial fibrillation, it’s important to live a heart-healthy lifestyle […] which may include increasing your physical activity […]. These snippets represent only an overview of over three pages of descriptive features available on atrial fibrillation.

#### MONDO disease ontology

Since the MONDO Disease Ontology^44^ harmonizes diseases from a wide range of ontologies, including the Online Mendelian Inheritance in Man (OMIM)^49^, SNOMED Clinical Terms (CT), International Classification of Diseases (ICD), and Medical Dictionary for Regulatory Activities (MedDRA), it was our preferred ontology for defining diseases. We retrieved the ontology from http://purl.obolibrary.org/obo/MONDO.obo on 31 May 2021. Processing involved parsing the ontology file to extract disease terms in the ontology, parent-child relationships, subsets of diseases, cross references to other ontologies, and definitions of disease terms. The processed data contains 64,388 disease-disease edges.

#### Orphanet

Orphanet^48^ is a database that focuses on gathering knowledge about rare diseases. The Orphanet resource at https://www.orpha.net/consor/cgi-bin/Disease_Search_List.php?lng=EN has curated information about definitions, prevalence, management and treatment, epidemiology, and clinical description for 9,348 rare diseases. We retrieved the resource data and extracted disease features on 10 May 2021 using the *orpha.py* script available in the PrimeKG repository.

Let us illustrate features in PrimeKG for rare Hurler syndrome with the Orphanet ID 93473. Hurler syndrome is the most severe form of mucopolysaccharidosis type 1, a rare lysosomal storage disease characterized by skeletal abnormalities, cognitive impairment, heart disease, […] and reduced life expectancy. The prevalence of the Hurler subtype of MPS1 is estimated at 1/200,000 in Europe and one in a million in general. The clinical manifestation of the disease includes ‘ musculoskeletal alterations, cardiomyopathy, […] and neurosensorial hearing loss within the first year of life. Management of the disease is multidisciplinary: ‘Hematopoietic stem cell transplantation is the treatment of choice as it can prolong survival. […] Enzyme replacement therapy (ERT) with laronidase […] is a lifelong therapy which alleviates nonneurological symptoms.’. These descriptions represent a brief snapshot of expertly curated knowledge incorporated in PrimeKG.

#### Four sources of physical protein-protein interactions

Protein-protein interactions (PPIs) are composed of experimentally-verified interactions between proteins. The interactions we consider are diverse in nature and include signaling, regulatory, metabolic-pathway, kinase-substrate and protein complex interactions, which are considered unweighted and undirected. We use the human PPI network compiled by Menche *et al*.^87^ as the starting resource. This resource integrates several protein-protein interaction databases, including TRANSFAC for regulatory interactions^88^, MINT and IntAct for yeast to hybrid binary interactions^89,90^, and CORUM for protein complex interactions^91^. Additionally, we retrieve protein-protein interaction information from from BioGRID^92^ and STRING^93^ databases. We also consider the human reference interactome (HuRI) generated by Luck *et al*.^94^, specifically, we use the high throughput investigation (HI) union, a combination of HuRI and several related efforts to systematically screen for protein-protein interactions. The processed data contains 642,150 edges.

#### Reactome pathway database

Reactome^95^ is an open-source, curated database for pathways. We retrieved information about pathways from https://reactome.org/download/current/ReactomePathways.txt, relationships between pathways from https://reactome.org/download/current/ReactomePathwaysRelation.txt and pathway-protein relations from https://reactome.org/download/current/NCBI2Reactome.txt on 31 May 2021. Processing involved extracting ontology information such as hierarchical relationships and extracting pathway-protein interactions. The processed data contains 5,070 pathway-pathway and 85,292 protein-pathway edges.

#### Side effect knowledgebases

The Side Effect Resource (SIDER)^96^ contains data about adverse drug reactions. We retrieved side-effect data (SIDER 4.1 version) from http://sideeffects.embl.de/media/download/meddra_all_se.tsv.gz and SIDER’s drug to Anatomical Therapeutic Chemical (ATC) classification mapping from http://sideeffects.embl.de/media/download/drug_atc.tsv on 31 May 2021. Processing involved extracting all side effects where the MedDRA term was coded at the preferred term level and then mapping drugs from STITCH identifiers^97^ to ATC identifiers. The processed data 202,736 contains drug-phenotype associations.

#### Uberon multi-species anatomy ontology

Uberon^98^ is an ontology that contains information about the human anatomy. We retrieved the ontology from http://purl.obolibrary.org/obo/uberon/ext.obo on 31 May 2021. Processing involved extracting information about anatomy nodes and the relationships between them. The processed data contains 28,064 hierarchical relationships between anatomy nodes.

#### UMLS knowledgebase

The Unified Medical Language System (UMLS) Knowledge Source^46^ contains information about biomedical and health-related concepts. We retrieved the complete UMLS Metathesauras from https://download.nlm.nih.gov/umls/kss/2021AA/umls-2021AA-metathesaurus.zip on 31 May 2021 in ‘.RRF’ format. To map UMLS CUI terms to the MONDO Disease Ontology, we used the ‘MRCONSO.RRF’ to extract UMLS Concept Unique Identifier (CUI) terms in English. We mapped UMLS CUI terms to MONDO terms in two ways. Firstly, we directly extracted cross-references between the two from the MONDO ontology. We indirectly mapped UMLS to MONDO using OMIM, National Cancer Institute Thesaurus (NCIT), MESH, MedDRA, ICD 10, and SNOMED CT as intermediate ontologies.

Further, we used ‘MRSTY.RRF’ and ‘MRDEF.RRF’ files to extract definitions for UMLS terms. Of the 127 semantic types in the ‘MRSTY.RRF’ file, we selected 11 that belonged to the Disorder semantic group in a manner consistent with prior work^99^. These semantic types were congenital abnormality, acquired abnormality, Injury or poisoning, pathologic function, disease or syndrome, mental or behavioral dysfunction, cell or molecular dysfunction, experimental model of disease, signs and symptoms, anatomical abnormality, and neoplastic process. We then used the ‘MRDEF.RRF’ file to extract definitions for CUI terms from sources that were in English.

#### Additional vocabularies

We retrieved gene names and mappings between NCBI Entrez IDs and UniProt IDs from https://www.genenames.org/download/custom/ on 31 May 2021. We retrieved the DrugBank drug vocabulary from https://go.drugbank.com/releases/5-1-8/downloads/all-drugbank-vocabulary on 31 May 2021. These were used to map nodes in PrimeKG to consistent ontologies.

### B. Standardizing and harmonizing data resources

To harmonize these primary resources into PrimeKG, we selected ontologies for each node type, harmonized datasets into a standardized format, and resolved overlap across ontologies. The process of defining node types and selecting common ontologies is illustrated in Fig. 2a where primary data records are colored if they are used to define unique identifiers for a node type. In the remainder of this study, we interchangeably refer to ‘gene/protein’ nodes as proteins and ‘effect/phenotype’ nodes as phenotypes. We mapped the aforementioned processed resources to ensure that all nodes were defined using unique identifiers from their respective ontologies and databases. Next, we identified sources of information across different primary resources for each node type to maximize the number of relationships in PrimeKG (Fig. 2b).

#### Resolving overlap between phenotype and disease nodes

Since both the MONDO Disease Ontology^44^ and Human Phenotype Ontology^45^ were developed by the Monarch Initiative, there exists a considerable overlap between phenotype nodes and disease nodes across the various datasets. Overlapping nodes were defined as effect/phenotype nodes in HPO that (i) had the same ID number as disease nodes in MONDO and (ii) could be mapped from HPO to MONDO using cross-references found in MONDO. These overlapping phenotype nodes were converted to disease nodes by manipulating edges in various datasets to avoid duplicate nodes. Let us define the set of overlapping phenotype nodes as *P*. Phenotype-phenotype edges extracted from HPO were converted to phenotype-disease edges if one phenotype node was in *P* and to disease-disease edges if both phenotype nodes were in *P*. Protein-phenotype edges extracted from DisGeNet^78^ were converted to protein-disease relations if the phenotype node was in *P* and removed from the group of protein-phenotype edges. Finally, for disease-phenotype and drug-phenotype relations, we dropped any edges where the phenotype was in *P*. Adding these edges to drug-disease relations would only introduce unnecessary noise to the indication, contraindication, and off-label use edges.

### C. Building precision medicine knowledge graph (PrimeKG)

To create PrimeKG’s graph, we merged the harmonized primary data resources into a graph and extracted its largest connected component as shown in Fig. 2c. We integrated the various processed, curated datasets and cleaned the graph by dropping NaN and duplicate edges, adding reverse edges, dropping duplicates again, and removing self-loops. This version of the knowledge graph is available in PrimeKG’s repository as ‘kg_raw.csv’. To ensure that PrimeKG is well-connected and has no isolated pockets, we extracted its largest connected component using the iGraph package^100^. Intuitively, extracting the largest connected component of the knowledge graph excludes nodes without edges connecting them to the rest of the graph. This giant component retained 99.998% of the edges that were present in the original graph. The largest connected component is available in PrimeKG’s repository as ‘kg_giant.csv’.

### D. Supplementing drug nodes with clinical information

We extracted both textual and numerical features for drug nodes in the knowledge graph from DrugBank^80^ and Drug Central^83^ (Fig. 2d). Features from DrugBank mapped directly to the knowledge graph since drugs were coded using DrugBank identifiers. Some features had unique attributes for each drug, such as ‘state’, ‘indication’, and ‘mechanism of action’, and others had numerous attributes for each drug, such as ‘group’ and ‘Anatomical Therapeutic Chemical (ATC) classification level’. The latter set of features was converted to single text descriptions by joining features using conjunctions such as ‘;’ and ‘and’. Features in Drug Central were mapped to DrugBank IDs using their Chemical Abstracts Service Registry (CAS) identifiers from the vocabulary retrieved from DrugBank. Once all features were mapped, text processing removed all tokens referenced in DrugBank (for example, “[L64839]”) with the help of regular expressions. We nullified locations where the text mentioned that no data was available for the half-life feature. Finally, we converted numerical features into textual descriptions in order to standardize the feature set.

We select the drug Prednisolone as an example to illustrate the depth of clinical information available in these features. The ‘[…]’ is used to compress text sections for brevity. Prednisolone is a glucocorticoid similar to cortisol used for its anti-inflammatory, immunosuppressive, anti-neoplastic, and vasoconstrictive effects. Prednisolone has a plasma half-life of 2.1–3.5 hours. Prednisolone is indicated to treat endocrine, rheumatic, and hematologic disorders; […] and other conditions like tuberculous meningitis. Corticosteroids binding to the glucocorticoid receptor mediates changes in gene expression that lead to […]. Prednisolone’s protein binding is highly variable […]. Corticosteroids bind to the glucocorticoid receptor, inhibiting pro-inflammatory signals and promoting […]. Prednisolone is solid. Prednisolone is part of Adrenal Cortex Hormones; Adrenals; […] Prednisolone is approved, and vet approved. Prednisolone uses Prednisone Action Pathway […] The molecular weight is 360.45. Prednisolone has a topological polar surface area of 94.83. The log p value of Prednisolone is 1.42.

### E. Supplementing disease nodes with clinical information

We extracted textual features for diseases nodes in the knowledge graph from the MONDO Disease Ontology^44^, Orphanet^48^, Mayo Clinic^55^, and UMLS knowledgebase^46^ (Fig. 2d). Features from all these sources were mapped to the ‘node_id’ field of disease nodes, which was defined using the MONDO Disease Ontology. Since disease nodes were grouped as described in the Technical Validation section, many diseases defined in the MONDO Disease Ontology (i.e., many ‘node_id’ values) were collapsed into a single node (i.e., unique ‘node_index’ values). Since disease features are mapped to MONDO identifiers or the ‘node_id’ field, it is possible for a single disease node in the knowledge graph, defined by a unique ‘node_index’, to have multiple feature values for a given feature. PrimeKG provides these features in their entirety.

Disease definitions from the MONDO Disease Ontology were directly extracted from the ontology file and unique for each ‘node_id’. Disease descriptions extracted from UMLS were mapped from Concept Unique Identifier (CUI) terms to MONDO and, as a result, numerous for each ‘node_id’. Using regular expressions, we removed tokens that were references and URLs from UMLS disease descriptions. From Orphanet, we extracted definitions, prevalence, epidemiology, clinical description, and management and treatment. We mapped the features from Orphanet IDs to MONDO, and as a result, there were multiple for each ‘node_id’. We used regular expressions to fix formatting errors in the prevalence and epidemiology features.

We extracted the following disease features from the Mayo Clinic’s knowledgebase: symptoms, causes, risk factors, complications, and prevention. Since the Mayo Clinic web-scrapping did not provide a unique identifier in any ontology, we mapped disease names in Mayo Clinic to those in the MONDO Disease Ontology. To develop this mapping, we used a strategy for grouping disease names described in detail in the Technical Validation section. Briefly, we conducted automated string matching followed by manual approval of all disease name mappings based on their Bidirectional Encoder Representations from Transformers (BERT) model^101^ embedding similarity. Automated string matching involved approving exact matches and encapsulating matches, where the name in Mayo was completely present in the name in MONDO. During processing the symptoms feature, we used regular expressions to extract the end of the text description that explained when to see the doctor as a new and separate feature. Finally, we fixed formatting errors in the text.

To illustrate the depth and breadth of information covered by the disease features, we select Hepatic Porphyria. The ‘[…]’ notation is used to compress sections of text for brevity. Per the MONDO Disease Ontology, Hepatic Porphyria is a group of metabolic diseases due to deficiency of one of a number of liver enzymes in the biosynthetic pathway of heme. They are characterized by […]. Clinical features include […]. The UMLS has a very similar disease description. According to Orphanet, it’s a rare sub-group of porphyrias characterized by neuro-visceral attacks with […]. In most European countries, the prevalence of acute hepatic porphyrias is around 1/75000. In 80% of cases, the patients are female. All acute hepatic porphyrias can be accompanied by neuro-visceral attacks that appear as […]. The attacks are most commonly triggered by […]. When an acute attack is confirmed, urgent treatment with an injection of […]. According to Mayo Clinic, signs and symptoms of acute porphyria may include severe abdominal pain, […], and seizures. All types of porphyria involve a problem in producing heme […], and a shortage of a specific enzyme determines the type of porphyria. In addition to genetic risks, environmental factors may trigger the development of […]. Examples of triggers include exposure to sunlight, […]. Possible complications depend on […] During an attack, you may experience […] Although there’s no way to prevent porphyria if you have the disease, avoid […]. When to see a doctor, […].

## Data Record

The Precision Medicine Knowledge Graph (PrimeKG) is available at Harvard Dataverse^102^. To develop PrimeKG, we retrieved and collated 20 data resources (detailed in the Methods section) as visualized in Fig. 2a, identified relations across these resources as shown in Fig. 2b,c, harmonized them into a heterogeneous network illustrated in Fig. 2c, and augmented the drug and disease nodes in the network with clinical features depicted in Fig. 2d. Language descriptions of drugs and clinical characteristics of diseases give the features of drug or disease nodes.

PrimeKG is a multimodal knowledge graph with 10 types of nodes, 30 types of undirected edges, and natural language descriptions for disease and drug nodes. PrimeKG contains 129,375 nodes and 4,050,249 edges. Figure 1a shows a schematic overview of the graph structure. We provide a breakdown of the number of nodes by node type and the number of edges by edge type in Tables 1, 2, respectively.

**Table 2:** Statistics on edges in PrimeKG.

Relation type	Count	Percent (%)
Anatomy - Protein (present)	3,036,406	37.5
Drug - Drug	2,672,628	33.0
Protein - Protein	642,150	7.9
Disease - Phenotype (positive)	300,634	3.7
Biological process - Protein	289,610	3.6
Cellular component - Protein	166,804	2.1
Disease - Protein	160,822	2.0
Molecular function - Protein	139,060	1.7
Drug - Phenotype	129,568	1.6
Biological process - Biological process	105,772	1.3
Pathway - Protein	85,292	1.1
Disease - Disease	64,388	0.8
Drug - Disease (contraindication)	61,350	0.8
Drug - Protein	51,306	0.6
Anatomy - Protein (absent)	39,774	0.5
Phenotype - Phenotype	37,472	0.5
Anatomy - Anatomy	28,064	0.3
Molecular function - Molecular function	27,148	0.3
Drug - Disease (indication)	18,776	0.2
Cellular component - Cellular component	9,690	0.1
Phenotype - Protein	6,660	0.1
Drug - Disease (off-label use)	5,136	0.1
Pathway - Pathway	5,070	0.1
Exposure - Disease	4,608	0.1
Exposure - Exposure	4,140	0.1
Exposure - Biological process	3,250	<0.1
Exposure - Protein	2,424	<0.1
Disease - Phenotype (negative)	2,386	<0.1
Exposure - Molecular function	90	<0.1
Exposure - Cellular component	20	<0.1
Total	8,100,498	100.0

Listed are the numbers of directed edges in PrimeKG.

Tables 3, 4 show statistics on the number of features available for drug and disease nodes. Disease features include the disease prevalence information, symptoms, causes, risk factors, epidemiology, clinical description, management and treatment, complications, prevention, and when to see a doctor. Drug features include molecular weight of chemical compounds, indications, mechanisms of action, pharmacodynamics, protein binding events, and pathway information. Figure 1c provides an example of the supporting information available across these features.

**Table 3:** Statistics on drug features in PrimeKG.

Source	Type of feature	Count	Unique	Percent (%)
Drug Central^83^	Molecular weight	2,797	2,308	35.2
	TPSA	2,718	2,718	34.2
	cLogP	2,574	980	32.3
DrugBank^80^	Group	7,957	7,903	100.0
	State	6,517	6,463	81.9
	Category	5,431	5,431	68.3
	Description	4,591	4,565	57.7
	Indication	3,393	3,076	42.6
	Mechanism of action	3,242	3,161	40.7
	ATC 4	2,818	1,040	35.4
	ATC 3	2,818	2,818	35.4
	ATC 2	2,818	2,818	35.4
	ATC 1	2,818	2,818	35.4
	Pharmacodynamics	2,659	2,617	33.4
	Half life	2,063	1,893	25.9
	Protein binding	1,669	1,487	21.0
	Pathway	598	598	7.5

The count column refers to the number of features including duplicates, and the Unique column refers to the number of unique features.

**Table 4:** Statistics on disease features in PrimeKG.

Source	Type of feature	Unprocessed KG		Processed KG	
		Count	Unique	Count	Unique
Combined	Combined	40,068	18,152	39,800	14,252
MONDO Disease Ontology^44^	Definition	15,238	15,238	15,238	12,001
UMLS^46^	Description	28,468	8,689	25,374	6,964
Orphanet^48^	Definition	6,564	6,548	6,562	5,645
	Prevalence	3,989	3,989	3,500	3,430
	Epidemiology	2,350	2,348	2,335	2,026
	Clinical description	2,294	2,292	2,293	1,972
	Management and treatment	1,732	1,731	1,722	1,553
Mayo Clinic^55^	Symptoms	6,642	5,789	5,140	4,470
	Causes	6,629	5,776	5,128	4,459
	Risk factors	6,284	5,501	4,898	4,299
	Complications	5,011	4,455	3,792	3,396
	Prevention	2,529	2,273	1,907	1,776
	When to see a doctor	5,862	5,234	4,531	4,058

Unprocessed KG refers to the initial knowledge graph assembled from datasets. Processed KG refers to the fully processed PrimeKG, and includes disease groupings. The count column refers to the number of features including duplicates, and the Unique column refers to the number of unique features. Note that the ‘Combined’ row has counts greater than the total number of diseases in the knowledge graph. This is not a discrepancy but rather reflects the complexity of the data involving missingness for some diseases and multiple descriptors for other diseases.

The PrimeKG knowledge graph has nodes of 10 types and uses the following terminologies and ontologies to describe the nodes. The node types ‘drug’, ‘disease’, ‘anatomy’ and ‘pathway’ are respectively encoded as terms in DrugBank^80^, MONDO^44^, UBERON multi-species anatomy ontology^98^, and Reactome pathway database^95^. Genes and proteins are treated as a single node type, ‘gene/protein’, and identified by Entrez Gene IDs^84^. The node types ‘biological process’, ‘molecular function’, and ‘cellular component’ are defined using Gene Ontology (GO) terms^86^. Disease phenotypes extracted from Human Phenotype Ontology (HPO)^45^ and drug side effects extracted from Side Effect Knowledgebase (SIDER)^96^ are collapsed into a single node type, ‘effect/phenotype,’ that is encoded using HPO IDs. Finally, ‘exposure’ nodes are defined using the ExposureStressorID field, which contains Medical Subject Headings (MeSH) provided by the Comparative Toxicogenomics Database (CTD)^81^.

The datasets in PrimeKG are structured to follow a standardized format. In the following, quotations indicate column names used to define PrimeKG. For each node in the knowledge graph, we provide ‘node_index’, which is a unique index to identify the node in PrimeKG; ‘node_id’, which indicates the identifier of the node from its ontology; ‘node_type’, which indicates the node type (Table 1); ‘node_name’ which indicates the name of the node as provided by the ontology; and ‘node_source’ which indicates the ontology from which ‘node_id’ and ‘node_name’ fields were extracted. For each edge in PrimeKG, we provide ‘relation’, which is the name of the edge type that connects the two nodes (Table 2); ‘x_index’, which links to the ‘node_index’ field; and ‘y_index’, which also links to ‘node_index’.

## Technical Validation

As part of the technical validation, we explore the structure and connectivity of PrimeKG.

### Distinguishing properties of PrimeKG

Here, we highlight four distinguishing properties of PrimeKG. We provide evidence and statistical support for these claims about PrimeKG in juxtaposition with three seminal biomedical knowledge graphs SPOKE^38^, HSDN^65^, and GARD^34^. Firstly, PrimeKG provides coverage for an extensive range of diseases. Our knowledge graph comprises 22,236 disease terms that are then grouped into 17,080 clinically meaningful diseases. Compared to existing graphs such as SPOKE, HSDN, and GARD, PrimeKG provides an improvement in disease coverage by one to two orders of magnitude. Next, while these existing resources primarily contain “treats” or indication edges between drugs and diseases, PrimeKG provides more intricate relationships with indication, contraindication, and off-label use edges.

Moreover, PrimeKG provides incredible coverage of rare diseases while remaining integrated with the entire range of diseases. Orphanet^48^ is considered the definitive authority on rare diseases. Of 9,348 rare diseases in Orphanet, 90.8% are present in PrimeKG as disease nodes. Previously, knowledge graphs have either scarce coverage of rare diseases (e.g., SPOKE, HSDN) or an exclusive focus on them (e.g., GARD). PrimeKG embraces the entire range of conditions from rare to prevalent across its 22,236 disease terms. Finally, PrimeKG is multimodal and contains clinical features for drugs and diseases. Traditionally, knowledge graphs have been defined solely as relationships between their nodes. These graph relationships tend to encode biological and molecular information but lack medically relevant descriptions. PrimeKG integrates clinically meaningful text descriptions for drug and disease nodes. This multimodality of PrimeKG enables the fusion of medical and molecular knowledge.

### PrimeKG is easy to use and update

PrimeKG is available as a single set of triplets of source nodes, relations, and target nodes. This data structure is agnostic to computing preferences and can be read using any programming language. We use Harvard Dataverse^102^ to make PrimeKG available in a single user-friendly CSV file so that there is no need for the user to connect to any external databases. Although PrimeKG has millions of edges, it can be loaded into memory using a standard CPU in less than 5.18 seconds ± 51.9 milliseconds. Queries on the knowledge graph generally take less than 1 second. Once loaded, PrimeKG can be converted into a graph structure through various commonly used libraries (such as iGraph or NetworkX for Python). We also provide tutorials for getting started and links to community data loaders on our GitHub repository.

The provenance of PrimeKG can be easily tracked on Harvard Dataverse^102^. All our data curation and processing approaches are transparent, fully reproducible, and can be continually adapted as data resources evolve and new data become available. We have provided detailed instructions on “Building an updated PrimeKG” on our PrimeKG GitHub repository. Complete information is given here for (a) downloading primary data records, (b) processing data for each primary record along with script names and expected outputs, and (c) a ready-to-run Jupyter notebook to build an updated PrimeKG. After building an initial version of PrimeKG, users can update individual resources as necessary.

### A case study to evaluate the relevance of PrimeKG to the clinical presentation of autism

For downstream inferences made using PrimeKG to be conducive to studying human disease, disease nodes in PrimeKG would need to be medically relevant. To this end, we next analyze if PrimeKG’s representation of diseases strongly relates to their clinical presentation by carrying out a case study on autism spectrum disorder. We were motivated to investigate autism because it not only has incredible clinical heterogeneity^103–105^ but this heterogeneity has also been studied to identify clinically meaningful subtypes^56,57^. We gauged the relevance of disease nodes related to autism in PrimeKG in two steps: first, by performing the entity resolution for autism concepts across all relevant primary data resources (see Data Record), and second, by examining the relationship between these autism concepts and clinical subtypes of autism.

**Fig. 3:**
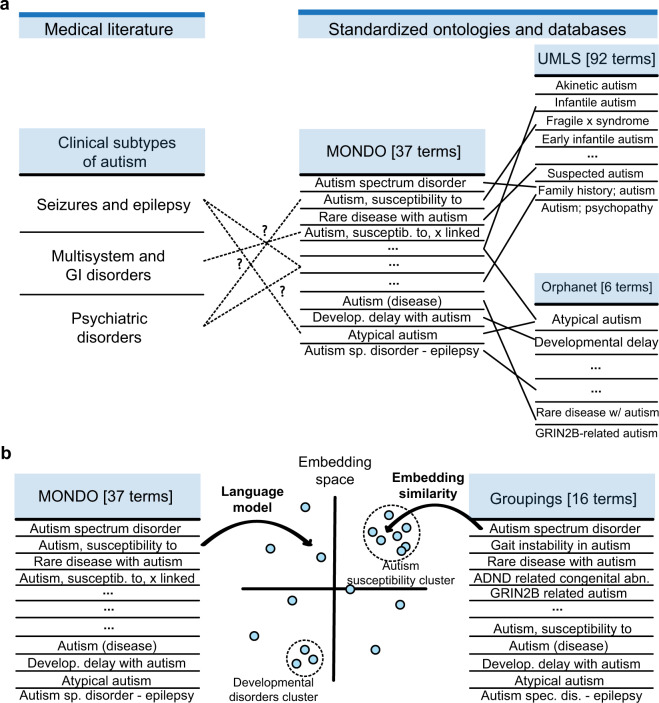
Reconciling autism disease nodes into clinically relevant entities. (**a)** The left side shows three clinically determined subtypes of autism. The right side shows autism-related disease terms across three ontologies: MONDO, UMLS, and Orphanet. While we can identify mappings across the ontologies, it is unclear how the terms in any ontology connect to clinical subtypes. (**b)** Illustration on how we use a language model, ClinicalBERT, to map terms from MONDO into a latent embedding space. Because the language model can group synonyms in the embedding space, we can cluster MONDO terms with similar semantic and medical meanings by calculating cosine similarity between embeddings of disease concepts. These clusters are created to develop disease groupings, as shown on the right in panel b. Abbreviations - MONDO: MONDO disease ontology, UMLS: unified medical language system.

We start by exploring whether autism disease nodes in PrimeKG reconciled the variation in autism concepts across databases and ontologies. For example, as demonstrated in Fig. 3a, MONDO disease ontology has 37 disease concepts related to autism, whereas the UMLS has 192 autism-associated concepts, and Orphanet has 6 autism-associated concepts. Although it is not immediately clear how these concepts relate to each other, we cannot develop a coherent knowledge graph without establishing connections between them. To this end, we overcome this challenge by defining all nodes using the MONDO disease ontology and mapping all other vocabularies to diseases in MONDO as outlined in Fig. 3a.

Finally, before using MONDO disease concepts as disease nodes in PrimeKG, we need to assess whether autism disease concepts in MONDO correlate with clinical subtypes of autism. Autism has been shown to manifest as three clinical subgroups characterized primarily by seizures, multisystem and gastrointestinal disorders, and psychiatric disorders^57^. However, it was unclear how MONDO’s 37 autism disease concepts (Fig. 3a) relate to the three clinically defined subtypes. In addition, there were many disease concepts in autism, such as ‘Autism, susceptibility to, 1’, ‘Autism, susceptibility to, 2’, ‘Autism, susceptibility to, x-linked’, etc., with no apparent clinical meaning, suggesting that disease nodes in MONDO do not correspond one-to-one to clinical manifestation of autism. For this reason, we developed a strategy to group diseases from MONDO into medically relevant and coherent nodes in PrimeKG. We proceed with describing and evaluating that strategy.

### Computational approaches to grouping disease nodes

As demonstrated in our case study of autism, disease concepts in MONDO may not correlate well with medical subtypes. MONDO contains many repetitive disease entities with no apparent clinical correlation. For this reason, we were motivated to group diseases in MONDO into medically relevant entities. Ideally, we would have preferred leveraging expertise across various disease areas when grouping these concepts. However, this approach was time-consuming, expensive, and challenging to execute at scale. Further, disease sub-phenotyping is a relatively new paradigm, so we anticipated low consensus among medical experts on what constitutes a unique disease.

Since manually grouping diseases with expert supervision was not feasible, we took a semi-automated unsupervised approach to group disease concepts in PrimeKG. Advances in natural language processing, specifically the Bidirectional Encoder Representations from Transformers (BERT) model^101^, allowed us to study the similarity between disease concept names. We grouped disease concepts with nearly identical names into a single node with string matching and BERT embedding similarity^101,106–109^.

We identified disease groups using a string-matching strategy across disease names^110^. In this strategy, we selected a disease that ended with a number, a roman numeral, or any alphanumeric phrase with a length of less than 2, or ‘type’ as the second-last word. Once such a disease was selected, we extracted the primary disease phrase by dropping the ending and used this phrase to find matches. Matches included diseases with the same initial phrase and those containing all phrase words with no other words, regardless of word order. The words ‘type’ and ‘(disease)’ were ignored for the latter matching criteria. In this manner, we grouped disease concepts in MONDO with string matching.

We further tightened groupings identified using string matching by exploring word embedding similarities between disease names, which is visualized in Fig. 3b. In natural language processing, word embeddings have been widely and successfully used to resolve conflicting and redundant entities in an unsupervised manner^110–112^, and deep language models such as BERT^101^ can produce semantically meaningful word embeddings. Specifically, ClinicalBERT^113^ is a BERT language model that encodes medical notions of semantics because it has been pre-trained on biomedical knowledge from PubMed^114^ and discharge summaries from MIMIC-III^115^. We used ClinicalBERT to extract word embeddings for disease group names identified during string matching. We also defined the similarity between two disease names as the cosine distance between their ClinicalBERT embeddings. Then, after applying an empirically chosen cutoff of similarity ≥0.98, we manually approved the suggested disease matches and assigned names to the new groups. Finally, these groupings were applied to the knowledge graph.

Finally, 22,205 disease concepts in MONDO were collapsed into 17,080 grouped diseases, which has resulted in a higher average edge density across diseases and more clinically relevant disease nodes. We anticipate that PrimeKG is a powerful dataset with this grouping because disease representations are dense and robust.

### Systematic evaluation of PrimeKG

Given the substantial investment and time required to develop a novel drug, network analysis has long been used to identify opportunities for expanding the use of already available drugs^116^. We demonstrate that PrimeKG can help detect drug repurposing opportunities. For this validation, we systematically retrieved 40 novel therapies approved by the FDA since June 2021. PrimeKG contains information up to 1 June 2021, limiting data leakage from PrimeKG to these therapies.

Of these 40 recent FDA-approved therapies, we identified 11 repurposed drugs in PrimeKG as listed in Table 5 and the remaining were novel compounds. We identified the disease corresponding to the indication for each drug and conducted network proximity calculations between relevant drug-disease pairs. As a first step, we would expect that only a few drug-disease pairs would have direct indication edges between them. However, as shown in Table 5, only one pair has such an edge, confirming that there has been no temporal data leakage.

**Table 5:** PrimeKG can identify drug repurposing opportunities.

Drug	Disease	Shortest distance	Randomized distance (95% CI)	Adjusted P value
Ropeginterferon alfa-2b-njft	Acquired polycythemia vera	1	—	—
Tirzepatide	Type 2 diabetes mellitus	2	3.45 (3.40–3.49)	<0.01
Tezepelumab-ekko	Asthma	2	3.68 (3.63–3.73)	<0.01
Tapinarof	Psoriasis	2	3.98 (3.93–4.02)	<0.01
Faricimab-svoa	Macular degeneration	2	4.21 (4.17–4.26)	<0.01
Inclisiran	Familial hypercholesterolemia	2	4.61 (4.56–4.66)	<0.01
Maribavir	Cytomegalovirus infection	3	4.40 (4.36–4.45)	<0.01
Belzutifan	Von Hippel-Lindau	3	4.55 (4.50–4.59)	0.01
Ganaxolone	CDKL5 disorder	3	4.32 (4.27–4.37)	0.03
Pacritinib	Myelofibrosis	3	3.83 (3.78–3.89)	0.08
Tralokinumab-ldrm	Atopic dermatitis	3	3.69 (3.65–3.74)	0.19

We retrieved 11 drugs with new indications approved by the FDA in the year after PrimeKG was assembled. We conducted a network proximity analysis between the repurposed drug and its indicated disease. Only 1 of 11 pairs already has an indication edge in PrimeKG, confirming that there is no temporal data leakage. We computer the shortest path distances between repurposed drug and (i). indicated disease; and (ii). a sample of 1000 non-indicated diseases. For the later, we have reported the mean randomized shortest path distance with 95% confidence intervals. We applied a non-parametric statistical analysis to assess how often the indicated shortest path distance is greater than the random shortest path distance and applied a Bonferroni correction to obtain the significance. At a threshold of P ≤ 0.05, relevant drugs are much closer to the indicated diseases than expected by random chance in 8 out of 10 cases. This analysis demonstrates the utility of PrimeKG for drug repurposing.

We conducted network analysis on these pairs by studying the shortest path distance between the repurposed drug and indicated disease. For each drug, we conducted permutation analysis by sampling 1000 non-indicated diseases and calculating the mean randomized shortest path distance with 95% confidence intervals between these pairs. Further, we applied a non-parametric statistical analysis that tested the number of times the indicated shortest path distance was greater than the random shortest path distance and applied a Bonferroni correction to obtain the significance. At a threshold of *P* ≤ 0.05, relevant drugs are much closer to the indicated diseases than expected by random chance in 8 out of 10 cases (Table 5). For example, in the case of familial hypercholesterolemia, one would need to parse through only 3.4% of drugs in PrimeKG to find a positive hit. This analysis demonstrates the utility of PrimeKG for drug repurposing and provides additional evidence that PrimeKG is a high-quality dataset.

The potential uses of PrimeKG are vast. PrimeKG describes drug features on a deeper biological level and disease features on a deeper clinical level, which can be used to explain genotype-phenotype associations in terms of genes, pathways, or any other nodes in an extensive knowledge graph, like PrimeKG. Consequently, PrimeKG can be paired with deep graph neural networks^117^ to help identify disease biomarkers, characterize disease processes, hone disease classification, identify phenotypic traits, and repurpose drugs. With the implementation of machine learning functionality, we anticipate that PrimeKG and similar knowledge graphs will become critical tools in advancing precision medicine.

## Data Availability

The PrimeKG’s project website is at https://zitniklab.hms.harvard.edu/projects/PrimeKG. The code to reproduce results, together with documentation and tutorials, is available in PrimeKG’s GitHub repository at https://github.com/mims-harvard/PrimeKG. In addition, the repository contains information and Python scripts to build new versions of PrimeKG as the underlying primary resources get updated and new data become available. PrimeKG data resource is hosted on Harvard Dataverse under a persistent identifier 10.7910/DVN/IXA7BM^102^. We have deposited the knowledge graph and all relevant intermediate files at this repository.
